# Radiological findings of contrast-induced encephalopathy following cerebral angiography: A case report

**DOI:** 10.1097/MD.0000000000033855

**Published:** 2023-05-17

**Authors:** Bin Wu, Ling Zeng, Kaifa Peng, Xi Shao, Li Liu, Rongyong Man, Xianbi Tang, Yushi Zhong

**Affiliations:** a Department of Neurology, The First People’s Hospital of Huaihua, Huaihua, PR China; b The Advanced Stroke Centre of China, Huaihua, PR China.

**Keywords:** case report, cerebral angiography, contrast-induced encephalopathy, iodixanol

## Abstract

Contrast-induced encephalopathy (CIE) is a rare complication associated with the use of contrast media. New contrast agents make contrast complications increasingly rare. The diagnosis of CIE is challenging, particularly in patients with acute ischemic stroke. Neuroimaging results for patients with CIE can also be highly variable.

**Patient concerns:**

A 63-year-old man with severe internal carotid artery stenosis who experienced several symptoms, including dizziness, nausea, vomiting, fever, and blurred vision after being administered the contrast agent iodixanol.

**Diagnoses:**

Multiple CT and MRI brain scans were performed. After excluding other differential diagnoses such as electrolytes imbalance, hypo/hyperglycemia and other neurological emergencies such as cerebral hemorrhage, cerebral infarction, the final diagnosis of CIE was made.

**Intervention:**

Treatment consisted of adequate hydration, intravenous dexamethasone, mannitol, and anticonvulsants.

**Outcome:**

The patient demonstrated progressive neurological improvement, and recovered from all symptoms on the fifth day. Follow-up at 3 months shows a good prognosis for patients.

**Conclusion:**

Patients with CIE may have a high signal on diffusion-weighted imaging and a low signal on apparent diffusion coefficient brain MRI. This is similar to the MRI findings in acute stroke. This needs to be distinguished from acute cerebral infarction and suggests that we should closely monitor patients’ neurological symptoms at the time of cerebral angiography and after the investigations.

## 1. Introduction

Most contrast-related complications are mild and reversible, but some can have serious effects on patients and even cause patients to die.^[1]^ Contrast-induced encephalopathy (CIE) is a rare complication following an iodinated contrast agent’s intravenous or intra-arterial administration.

This article reports the changes in the CIE patient’s brain computed tomography (CT) and magnetic resource imaging (MRI) performed after brain angiography with a new third-generation iso-osmolar nonionic contrast agent called iodixanol.

## 2. Case report

On a carotid artery ultrasound, a 63-year-old man was found to have severe stenosis of the internal carotid artery. He had a medical history of lower extremity vein thrombosis. Oral anticoagulation therapy with “Rivaroxaban tablets.” Deny history of food and drug allergies. Denied history of smoking and alcoholism. Physical examinations were generally normal and neurological examination was negative. Our outpatient carotid vascular ultrasound showed severe stenosis (70–99%) at the beginning of the right common carotid artery. He underwent cerebral angiography and received a total of 100 mL of iodixanol (Visipaque 320). This was the patient’s first exposure to a contrast agent.

**Figure 1.**
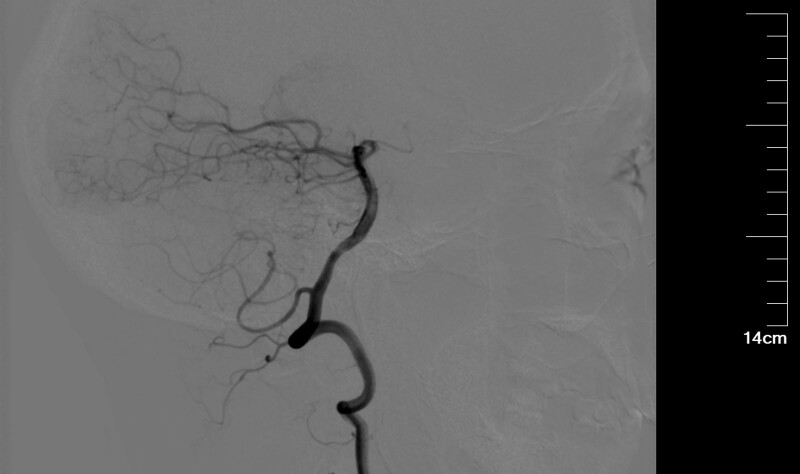
Imaging in the cerebral angiography. There was no narrowing or occlusion of the right posterior cerebellar artery.

**Figure 2.**
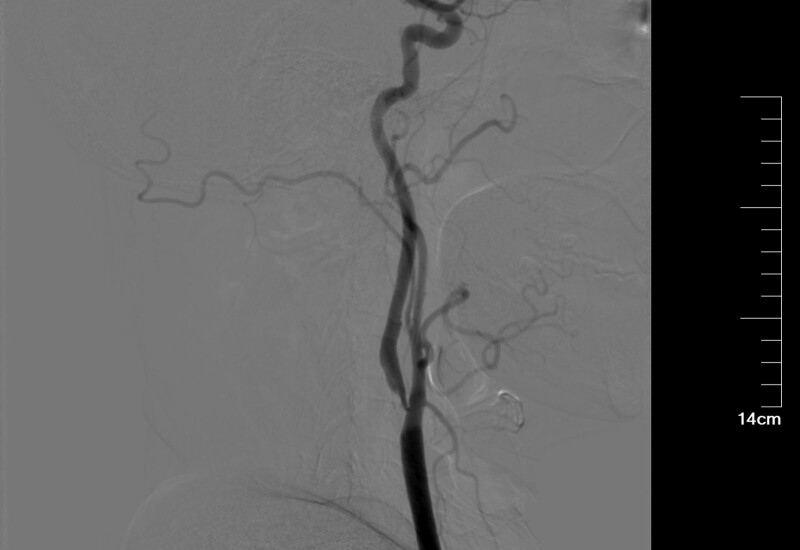
Imaging in the cerebral angiography. Common carotid artery (CCA) angiography revealed severe stenosis at the bifurcation of the right carotid artery.

The cerebral angiogram shows no stenosis or occlusion on the right posterior inferior cerebellar artery (Fig. 1). However, stroke-like changes in the area of vascular innervation were seen on MRI of the head several hours later. Cerebral angiography shows severe stenosis at the beginning of the right internal carotid artery (Fig. 2). The patient felt dizzy and nauseous during the procedure. After returning to the ward, the patient presented profuse sweating, nausea, and vomiting, accompanied by chest tightness, shortness of breath, fever, blurred vision, and slurred speech. Physical examination revealed spontaneous horizontal nystagmus. Brain CT and MRI were performed 2 hours after production.

**Figure 3.**
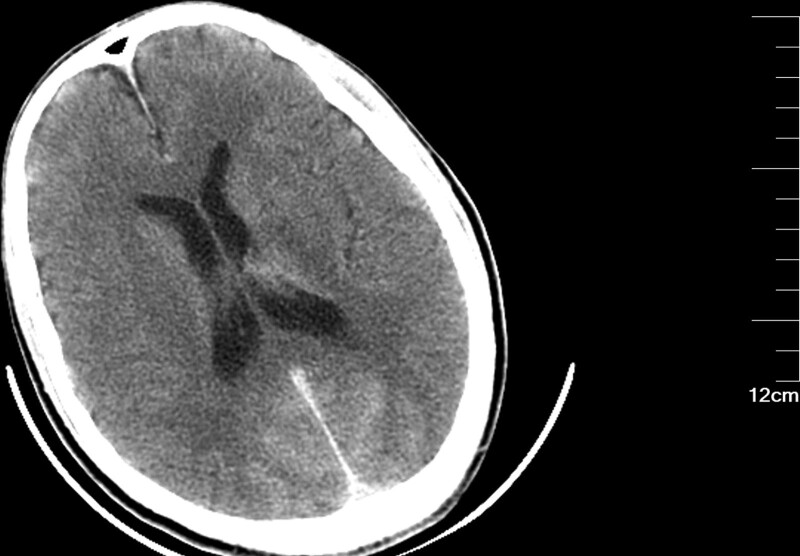
Imaging after cerebral angiography. The brain CT showed subarachnoid enhancement in the occipital region. CT = computed tomography.

**Figure 4.**
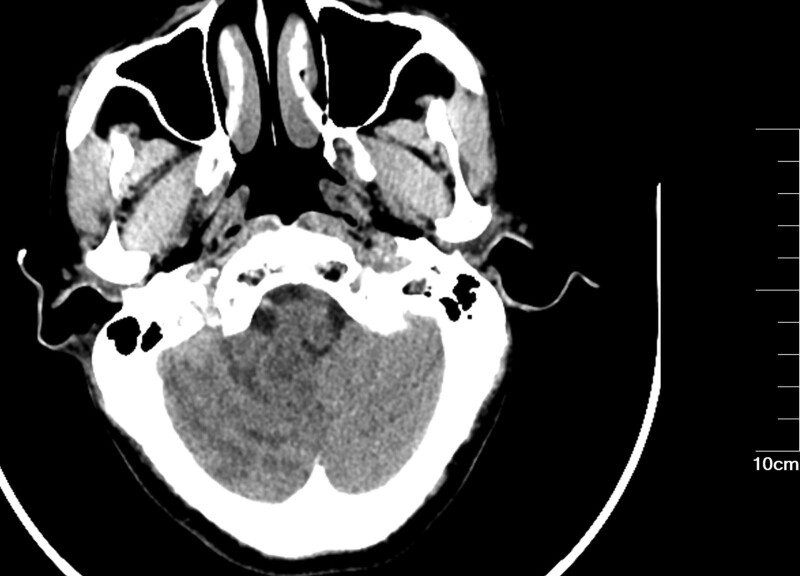
Follow-up CT 5 days later demonstrated a hypointense image of the right cerebellum (Inferior posterior cerebellar artery blood supply area). CT = computed tomography.

**Figure 5.**
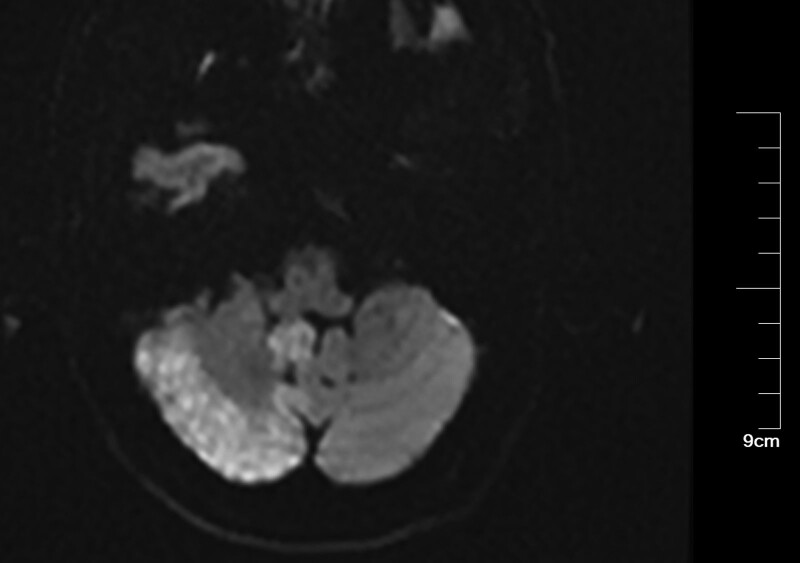
Imaging after cerebral angiography. DWI revealed a high-intensity lesion in the right cerebellum. DWI = diffusion-weighted imaging.

**Figure 6.**
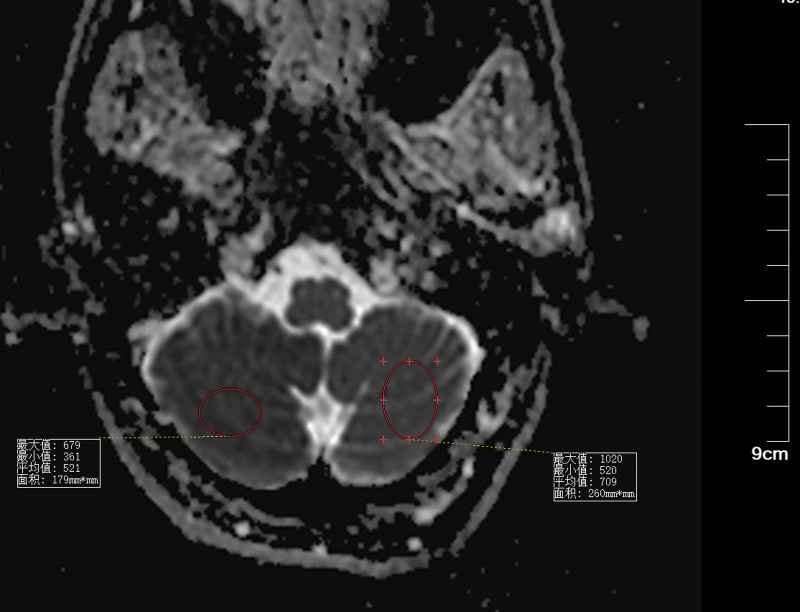
ADC suggests that the right cerebellar image signal (mean 521) is lower than the left normal image signal (mean 709). ADC = apparent diffusion coefficient.

**Figure 7.**
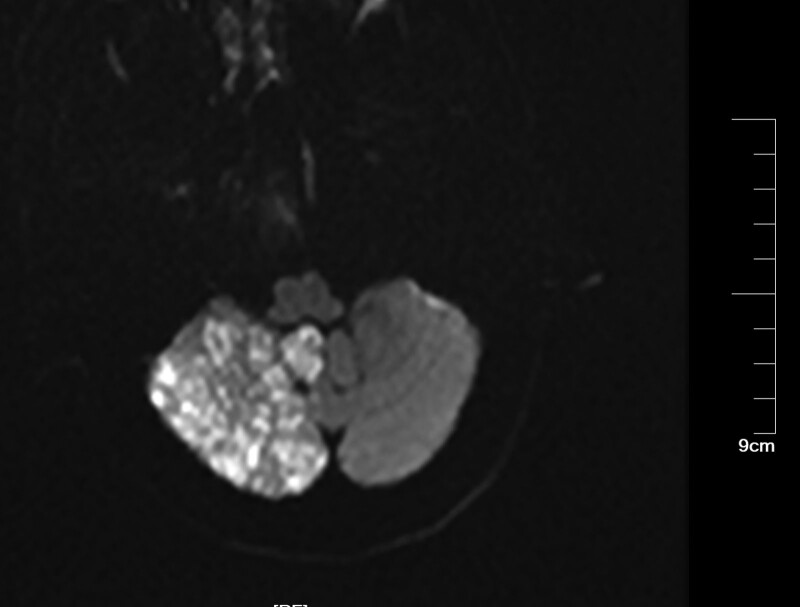
Follow-up DWI 2 days later demonstrated a significant high-intensity lesion in the right cerebellum. DWI = diffusion-weighted imaging.

**Figure 8.**
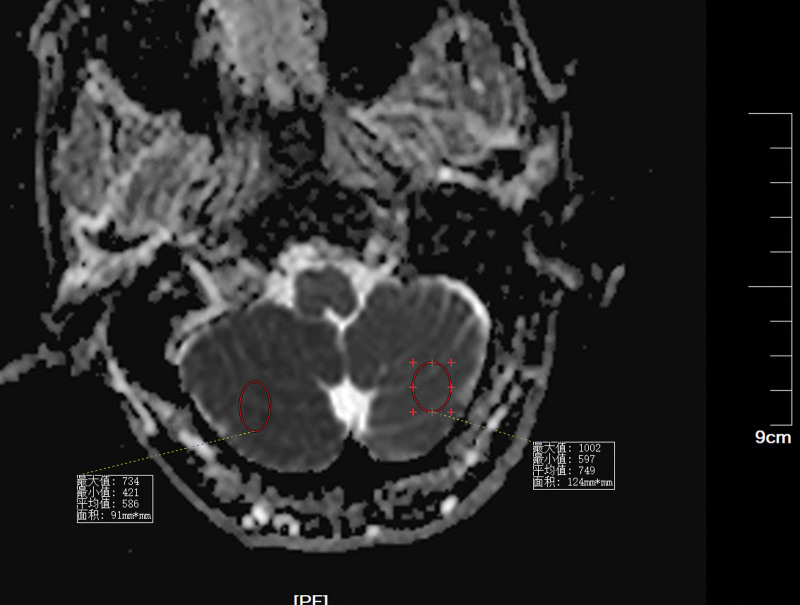
Follow-up ADC 2 days later demonstrated the right cerebellar image signal (mean 586) is lower than the left normal image signal (mean 749). ADC = apparent diffusion coefficient.

Patients with CIE may show abnormal cerebral edema and subarachnoid enhancement on head CT (Fig. 3). The follow-up CT 5 days later demonstrated a hypointense image of the right cerebellum (Fig. 4). MRI of the head can better aid in our diagnosis. In this patient, the diffusion-weighted imaging (DWI) image on the day of onset was heterogeneous with a high signal (Fig. 5), and the corresponding area on his apparent diffusion coefficient (ADC) image (Fig. 6) was a slightly low signal, and the corresponding area of the lesion was more pronounced on the second day (Figs. 7–8), which required differentiation from acute cerebral infarction.

**Figure 9.**
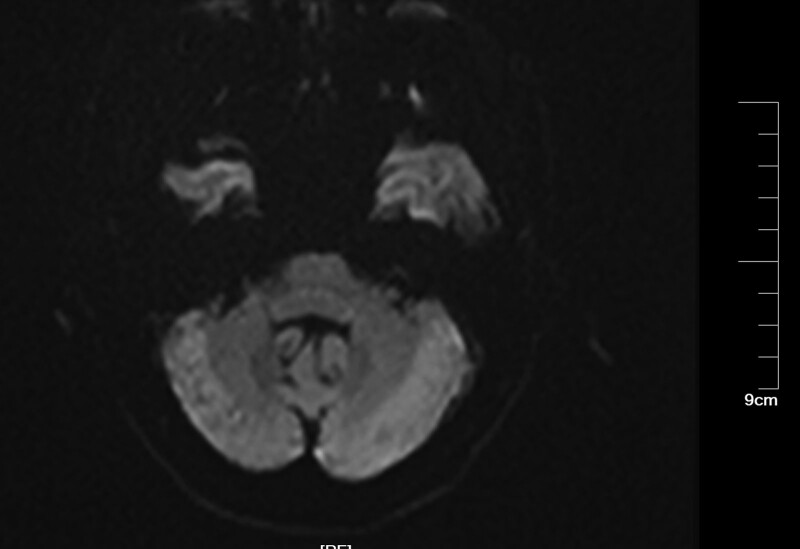
Follow-up imaging 3 months later showed normal brain images on both diffusion-weighted imaging (DWI) and apparent diffusion coefficient (ADC) scans.

**Figure 10.**
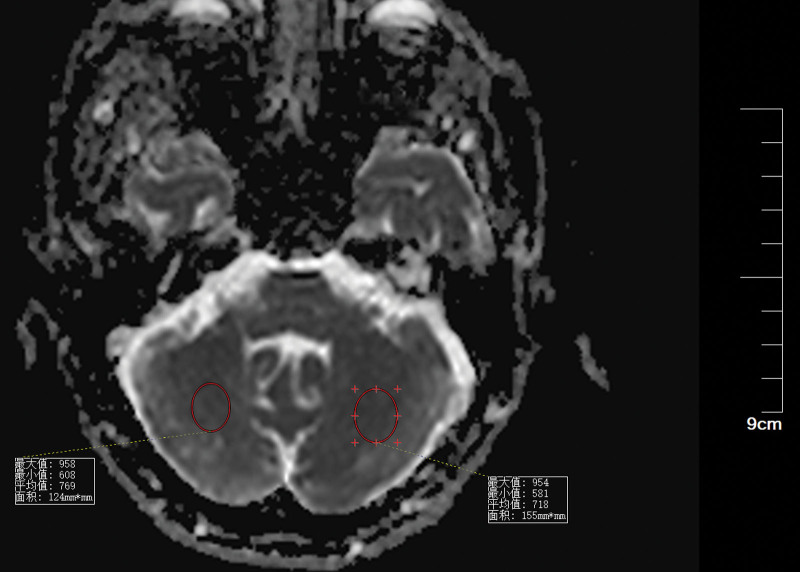
This indicates that the patient’s brain had recovered from any adverse effects of the contrast agent and that the treatment was effective in reducing the damage caused by CIE. CIE = contrast-induced encephalopathy.

The patient’s symptoms improved significantly within a few days without progressive deterioration. All of these lesions disappeared on the final follow-up head MRI (Figs. 9–10), indicating the presence of different imaging changes in CIE. Treatment for CIE typically involves symptomatic management and glucocorticoids to reduce the cellular inflammatory response. In this patient, hydration, intravenous dexamethasone, mannitol, and anticonvulsants were used, significantly improving symptoms within a few days. This case highlights the importance of recognizing and managing CIE, as well as the role of imaging in diagnosing and monitoring the disease.

## 3. Discussion

CIE is a rare complication of intravascular contrast media. Most CIE cases are reported after arterial contrast media applications, such as cerebral angiography.

Contrast agents are classified as ionic or nonionic according to their ability to ionize in solution, and as hypertonic, hypo-hyperosmolar or isotonic according to human plasma osmolality. Currently, nonionic contrast agents are widely used because they are significantly safer than ionic contrast agents. This article reports the changes in the CIE patient’s brain CT and MRI performed after brain angiography with a new third-generation iso-osmolar nonionic contrast agent called iodixanol. In this article, the patient presented symptoms of cerebellar neurological deficit with nausea and vomiting, fever, cortical blindness, and nystagmus.

The diagnosis of CIE is based on history and clinical presentation. It is usually distinguished from acute cerebrovascular accidents and reversible posterior encephalopathy syndromes. Head imaging can help in diagnosis.

Patients with CIE may show abnormal cerebral edema and subarachnoid enhancement on head CT. MRI of the head can better aid in our diagnosis, and a study showed^[2]^ that the lesions of CIE revealed a high intensity on DWI of MRI and is signal on ADC images, thus helping to aid in differentiation from acute cerebral infarction. Typical radiological findings include cerebral edema and cortical enhancement.^[3]^ Radiological signs, such as cerebral edema and cortical enhancement, play an important role in differentiating CIE from other neurological conditions. Various changes in the head CT images of the CIE patient, even with normal head CT findings.^[4]^ MRI findings of the head can help us diagnose CIE more effectively. On MRI, FLAIR hyperintense swollen cortical areas can be seen, and DWI is used to differentiate CIE from acute ischemia (restricted diffusion is seen in the latter but not in the former). At the same time, gyral swelling and hyperintensity on T2 FLAIR image and DWI, not accompanied by changes in ADC, have been described in the previously reported cases.^[5–7]^

The mechanism of CIE is not well understood, and there are 2 main theories. One is that the blood-brain barrier is disrupted. The occipital cortex is one of the most permeable regions of the blood-brain barrier, and contrast agents enter the skull with arterial blood flow and are easily deposited in the occipital region, causing toxic damage to cells and resulting in occipital cortical blindness.^[8]^ Most of the CIE seen by the author is related to the arterial use of contrast agents, and there is one report of CIE due to the intravenous use of contrast agents.^[9]^ This also supports the notion that the local concentration of contrast agents increases in the brain within a short period and poisons nerve cells through the blood-brain barrier. There is also the theory of cerebral vasospasm, in which the contrast agent rapidly fills the arterial vasculature after using a high-pressure gun for arterial angiography, and the higher vessel wall tension tends to cause arterial vasospasm, resulting in transient ischemia in the local arterial blood supply area.^[10]^ Pagani’s study supports this view.^[11]^ However, the duration of CIE usually ranges from hours to days and does not support the theory of pure vasospasm. The usual cerebral arterial vasospasm resolves in minutes to tens of minutes. Therefore, the mechanism of CIE needs to be further investigated.

At present, the risk factors for patients with CIE are unclear. Some studies have shown that hypertension, age, gender, contrast agent type, contrast agent dose and renal function are high-risk factors for CIE.^[10]^ However, in this article, the patient had no previous history of hypertension and normal renal function, but still developed CIE after using a safer third-generation iso-osmolar nonionic contrast agent, suggesting that CIE can also occur in patients with low-risk factors, which should alert clinicians. The clinical manifestations of CIE vary and usually include cortical blindness and vomiting,^[8]^ aseptic meningitis,^[12]^ and other symptoms of intracranial hypertension.^[13]^

However, the treatment of CIE requires a multidisciplinary approach and close monitoring of the patient’s neurological status. Prompt recognition of CIE and early initiation of treatment can improve the patient’s outcome. In this article, the patient was treated with hydration, intravenous dexamethasone, mannitol, and anticonvulsants, and he showed progressive neurological improvement and full recovery of his mental status within 5 days. Early and proper treatment of CIE can greatly reduce the risk of permanent neurological deficits. In conclusion, CIE is a rare but potentially serious complication of contrast media administration. Clinical awareness, early recognition, and prompt management are crucial to minimizing their impact on patients.

This article shows the changes in head imaging in patients with CIE at different times and highlights the importance of recognizing CIE as a potential complication following contrast media use and the need for prompt diagnosis and treatment. Further studies are needed to establish the safety and efficacy of different contrast agents, especially in high-risk populations such as elderly patients, those with underlying kidney disease, and those with a history of stroke. This will help improve patient outcomes and reduce the burden of CIE on healthcare systems.

## 4. Conclusions

Our report shows that even an iso-osmolar nonionic contrast agent, even iodixanol, can cause CIE. Therefore, CIE should be considered in the differential diagnosis of a patient with acute neurological symptoms after cerebral angiography, regardless of the type of contrast agent used. Neurological imaging is essential to rule out other more common and serious complications such as cerebral infarction or intracranial hemorrhage. Magnetic resonance imaging of the head in patients with contrast encephalopathy may show a high signal of DWI and a low signal of ADC, but the degree of low signal of ADC is milder than in cerebral infarction.

## Author contributions

**Conceptualization:** Ling Zeng, Yushi Zhong.

**Methodology:** Kaifa Peng, Xi Shao.

**Project administration:** Yushi Zhong.

**Supervision:** Li Liu, Xianbi Tang.

**Writing – original draft:** Bin Wu, Ling Zeng.

**Writing – review & editing:** Rongyong Man.
